# Lobectomy versus sublobar resection for stage I non-small cell lung cancer: an umbrella review of evidence quality, overlap, surgical-extent heterogeneity, and survival outcomes

**DOI:** 10.3389/fonc.2026.1836910

**Published:** 2026-06-05

**Authors:** Xiang Lin, Jingwen Zhang, Beinuo Wang, Zhenghao Dong, Yu Tong, Jian Zhou, Hu Liao

**Affiliations:** 1 Department of Thoracic Surgery, West China Hospital, Sichuan University, Chengdu, China; 2 West China School of Medicine, Sichuan University, Chengdu, Sichuan, China

**Keywords:** disease-free survival, lobectomy, minimally invasive surgery, non-small cell lung cancer, overall survival, segmentectomy, sublobectomy, umbrella review

## Abstract

**Background:**

Whether sublobectomy achieves survival outcomes comparable to lobectomy in Stage I non-small cell lung cancer (NSCLC) remains controversial. With increasing adoption of parenchymal-sparing surgery, a comprehensive evaluation of available evidence is warranted.

**Methods:**

A bibliometric analysis (2015–2025) was conducted alongside an umbrella review. PubMed, Embase, Web of Science, and CINAHL were searched for systematic reviews and meta-analyses comparing lobectomy and sublobectomy in Stage I NSCLC. Methodological quality was assessed using AMSTAR-2, and certainty of evidence was graded with GRADE. Summary hazard ratios (HRs) and heterogeneity were recalculated using random-effects models after removal of duplicate primary studies.

**Results:**

Eighteen reviews were included (9 high quality, 4 moderate, 5 critically low). Bibliometric analysis showed recent research bursts related to overall survival, disease-free survival, pulmonary segmentectomy, multicenter studies, and robotic-assisted thoracoscopic surgery. For overall survival (OS), pooled HRs varied and several analyses showed substantial heterogeneity (I² >70%). In recalculated pooled analyses, Stage I NSCLC showed an HR of 1.09 (95% CI 1.02–1.16; I² = 71.6%) and Stage IA an HR of 1.10 (95% CI 0.99–1.22; I² = 77.1%). For disease-free survival (DFS), HRs were 1.13 (95% CI 1.04–1.23; I² = 9.4%) for Stage I and 1.13 (95% CI 1.01–1.27; I² = 21.8%) for Stage IA. Evidence certainty was predominantly low or very low.

**Conclusion:**

Sublobar resection was associated with small but statistically significant higher hazards for OS and DFS in the broader Stage I population, whereas Stage IA OS did not differ significantly. However, OS estimates were limited by substantial heterogeneity, small-study effects, overlap among primary evidence, and predominantly low or very low certainty. This umbrella review clarifies the quality, bias, overlap, and applicability of existing meta-analytic evidence rather than replacing recent randomized trials.

**Systematic Review Registration:**

https://www.crd.york.ac.uk/PROSPERO/view/CRD420251150573, identifier CRD420251150573.

## 1 Introduction

Globally, lung cancer remains the primary cause of cancer-related mortality, characterized by rapid metastatic progression and poor prognostic outcomes, and non–small cell lung cancer (NSCLC) accounts for the majority of cases (1–3). High-resolution and low-dose helical CT screening have significantly increased the detection of early-stage NSCLC (4). Surgical resection remains the cornerstone of curative therapy for these operable tumors.

Lobectomy is the established standard of care and has been shown to reduce recurrence and improve long-term survival (5). However, growing interest in sublobar resection has reshaped contemporary discussions of surgical management in early-stage NSCLC. Wedge resection removes lung parenchyma containing the lesion without regard to anatomic boundaries, whereas segmentectomy involves resecting a defined anatomic segment and often entails more systematic lymph node dissection. Determining whether anatomic segmentectomy yields oncologic outcomes comparable to lobectomy has become a central focus among thoracic surgeons and oncologists. If oncologic equivalence is confirmed for small lesions, segmentectomy may be preferred given its advantages in lung function preservation and reduced morbidity, particularly for patients with limited pulmonary reserve or older adults (6). Nevertheless, the role of sublobar resection in lung cancer surgery remains an ongoing subject of debate.

This umbrella review applies a systematic approach to identify, appraise, and synthesize evidence from recent systematic reviews and meta-analyses comparing lobectomy and sublobectomy for stage I NSCLC. The primary objective is to clarify the comparative efficacy of the two procedures with respect to key oncologic outcomes, including overall survival (OS) and disease-free survival (DFS). Secondary aims include evaluating the methodological quality of existing meta-analyses, exploring variations in outcomes by tumor size, and identifying critical gaps to inform future research. Ultimately, this review aims to provide a structured synthesis to support evidence interpretation and individualized clinical decision-making.

## 2 Methods

### 2.1 Bibliometric analysis

This study retrieved relevant literature on non–small cell lung cancer (NSCLC) from both the Web of Science Core Collection (WOSCC) and PubMed (MEDLINE). For WOSCC, the Science Citation Index Expanded (SCI-EXPANDED) and Social Sciences Citation Index (SSCI) were searched. All records published between January 1, 2015, and September 30, 2025 were included. A high-sensitivity search strategy was applied to maximize the retrieval of potentially eligible studies, and no restrictions on tumor stage or study design were imposed during the initial search. The detailed search strategies for each database are provided in **Supplementary Table 1**. Bibliometric analyses were conducted using VOSviewer (version 1.6.20) (7) and CiteSpace (version 6.3.R1) (8).

### 2.2 Umbrella review

The protocols for this umbrella review were registered on two platforms: the International Platform of Registered Systematic Review and Meta-analysis Protocols and PROSPERO. This study adheres to the PRISMA guidelines for reporting systematic reviews and meta-analyses (9).

#### 2.2.1 Literature search strategy and eligibility criteria

We conducted a systematic search across four databases (PubMed, Web of Science, Embase, and CINAHL) for systematic reviews and meta-analyses comparing lobectomy and sublobar resection for early-stage lung cancer, particularly Stage I NSCLC. Studies published between January 1, 2015, and September 20, 2025, were included. Two researchers independently screened titles, abstracts, and full texts to identify studies potentially meeting the inclusion criteria. Any disagreements were resolved through discussion with a third reviewer. To minimize the risk of missing relevant studies, the reference lists of eligible articles were also manually searched. The detailed search strategy and the inclusion and exclusion criteria are presented in **Supplementary Tables 2** and **3**, respectively.

#### 2.2.2 Data extraction and estimate of methodological quality

Two researchers independently extracted data and assessed methodological quality for all eligible systematic reviews. A summary of the data extraction domains is provided in **Supplementary Table 6**. The following data were extracted: first author, publication year, country or region, number of primary studies, study design of primary studies, number of studies included in each systematic review or meta-analysis, sample size, study design, effect metrics, effect estimates with 95% confidence intervals (CIs), quality assessment tools, and authors’ conclusions. For each individual primary study, extracted data included the first author, publication year, study type, study design, sample size, outcome metrics, findings, and the maximum effect size reported in each meta-analysis.

For each article, the AMSTAR 2 checklist (‘A Measurement Tool to Assess Systematic Reviews, version 2’) was used to evaluate methodological quality (10). Any discrepancies between reviewers were resolved through discussion, and a third reviewer was consulted when necessary. The detailed assessments are presented in **Supplementary Table 4**.

#### 2.2.3 Overlapping and outdated reviews

Before synthesizing results, we assessed overlap among primary studies included in two or more reviews with the same exposure and outcome by constructing a citation matrix (11). We calculated the corrected covered area (CCA) and categorized overlap as very high (>15%), high (11–15%), moderate (6–10%), or minor (0–5%) according to established thresholds (11). The calculation formula was as follows:


$$\text{CCA}=\frac{N-r}{rc-r},$$

where *N* = total number of study occurrences,

*r* = number of unique studies,

*c* = number of meta-analyses.

Overlap among reviews was managed as follows:

1. For reviews with significant overlap (CCA ≥15%), priority was given to those with the highest AMSTAR 2 and GRADE ratings, the most recent publication date, those providing summary effect estimates or conducting meta-analyses, and those with the largest study or participant samples.
2. In cases of lower overlap (CCA<15%), both reviews were retained for comparison.
3. Duplicate and potentially overlapping primary studies were adjudicated using first author, publication year, study design, database source, recruitment period, sample size, stage criteria, and surgical comparison (11). For registry-based studies using SEER, NCDB, or other administrative datasets, potential overlap was further assessed by calendar years and eligibility criteria (11).

The deduplication and overlap adjudication rules are summarized in **Supplementary Table 11**.

#### 2.2.4 Data analysis

We re-estimated summary effect sizes, 95% confidence intervals (CIs), and P values for each meta-analysis using the DerSimonian and Laird method under a random-effects model. Additionally, to address methodological quality, meta-analyses were stratified according to AMSTAR 2 ratings and GRADE ratings to provide a more rigorous evaluation of evidence strength. Heterogeneity among included studies was assessed using Cochran’s Q test and the I² statistic, with I² >50% indicating substantial heterogeneity. Review-level exploratory trim-and-fill and leave-one-out sensitivity analyses were conducted using R with the metafor package; the extraction tables and analytic code are available from the corresponding author upon reasonable request.

We also calculated 95% prediction intervals, where intervals excluding the null value indicate a potential effect in future studies (12). Publication bias was evaluated using Egger’s test, with P< 0.10 indicating the presence of small-study effects (13, 14). The estimate from the largest study was compared with the random-effects estimate (13). Notably, the interpretation of prediction intervals, small-study effects, and excess significance bias applies only to analyses including three or more studies (15). Larger discrepancies between observed and expected values indicate greater significance bias (13).

We combined primary studies from previously published meta-analyses into recalculated pooled analyses after removing duplicated studies. Effect sizes for dichotomous outcomes were expressed as hazard ratios (HRs) or odds ratios (ORs) with corresponding 95% confidence intervals (CIs). Hazard ratios (HRs) with standard errors (SEs) were used for time-to-event outcomes. A P value< 0.05 was considered statistically significant. All analyses were performed using Python (version 3.12.5) with the statsmodels and scipy libraries. When available, subgroup information reported in the included meta-analyses, such as tumor size, age-related subgroup, surgical extent, or solid-dominant/GGO-dominant features, was extracted descriptively. Because this umbrella review was based on aggregate data from published reviews rather than individual patient-level datasets, *de novo* subgroup analyses by age or tumor size were not uniformly feasible. We classified the included reviews and extractable estimates as segmentectomy-only, wedge-only, mixed sublobar resection, or unclear sublobar definition. Reviews described only as “sublobar resection,” “limited resection,” or “sublobectomy” were not interpreted as segmentectomy-specific unless segmentectomy data were separately extractable (19, 22, 25, 30, 36). Review-level exploratory sensitivity analyses were performed to assess surgical-extent findings and publication-bias signals. These analyses included Egger’s test, Duval and Tweedie trim-and-fill, and leave-one-out sensitivity analyses when sufficient estimates were available. Because these analyses were based on review-level aggregate estimates rather than fully deduplicated primary-study-level effects, they were interpreted as exploratory. Surgical-extent classification and review-level exploratory sensitivity analyses are presented in **Supplementary Tables 10** and **12**.

#### 2.2.5 Certainty of evidence assessment

The credibility of the pooled evidence for each meta-analysis was evaluated using the GRADE (Grading of Recommendations, Assessment, Development and Evaluations) framework (16). GRADE assesses the certainty of evidence across five key domains:

1. Risk of bias,
2. Inconsistency,
3. Indirectness,
4. Imprecision, and
5. Publication bias.

For each outcome, the initial level of certainty was set based on study design (e.g., high for RCTs and low for observational studies). Downgrading was considered when serious or very serious concerns were present in any domain. The final rating for the certainty of evidence was categorized as high, moderate, low, or very low. GRADE ratings were independently assessed by two reviewers, and discrepancies were resolved through discussion or consultation with a third reviewer. The detailed assessment is outlined in **Supplementary Table 5**.

## 3 Results

### 3.1 Basic trends of research on surgical methods for stage I non-small cell lung cancer

**Figure 1:**
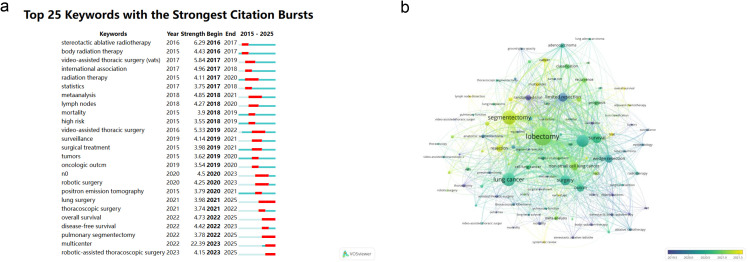
Keyword analysis of surgical research in stage I non–small cell lung cancer (NSCLC). **(a)** Top 25 keywords with the strongest citation bursts from 2015 to 2025; red bars indicate periods of increased research activity. **(b)** Keyword co-occurrence network generated by VOSviewer; node size reflects keyword frequency, link thickness indicates co-occurrence strength, and colors represent the average publication year.

As shown in **Figure 1a**, keyword burst analysis identified several periods of intensified research interest. Early hotspots (2015–2018) were primarily centered on radiation therapy, video-assisted thoracic surgery (VATS), and stereotactic ablative radiotherapy, reflecting the initial exploration of minimally invasive and non-surgical alternatives. Subsequently, from 2018 to 2021, attention shifted toward lymph nodes, mortality, and meta-analysis, indicating a growing emphasis on oncologic outcomes and evidence synthesis. More recently (2022–2025), emerging bursts such as overall survival, disease-free survival, pulmonary segmentectomy, multicenter, and robotic-assisted thoracoscopic surgery highlight a transition toward precision surgery, long-term prognostic evaluation, and high-quality multicenter evidence.

The keyword co-occurrence network (**Figure 1b**) further demonstrates the intellectual structure of this field. Core nodes such as lobectomy, segmentectomy, lung cancer, and survival occupy central positions, indicating that comparative effectiveness between standard lobectomy and sublobar resection remains the dominant research focus. Surrounding clusters include terms related to minimally invasive surgery (e.g., VATS, robotic surgery), oncologic outcomes (e.g., recurrence, prognosis, overall survival), and tumor characteristics (e.g., ground-glass opacity, tumor size), suggesting a multidisciplinary integration of surgical techniques, tumor biology, and outcome assessment.

### 3.2 Characteristics of studies included in umbrella review

**Figure 2:**
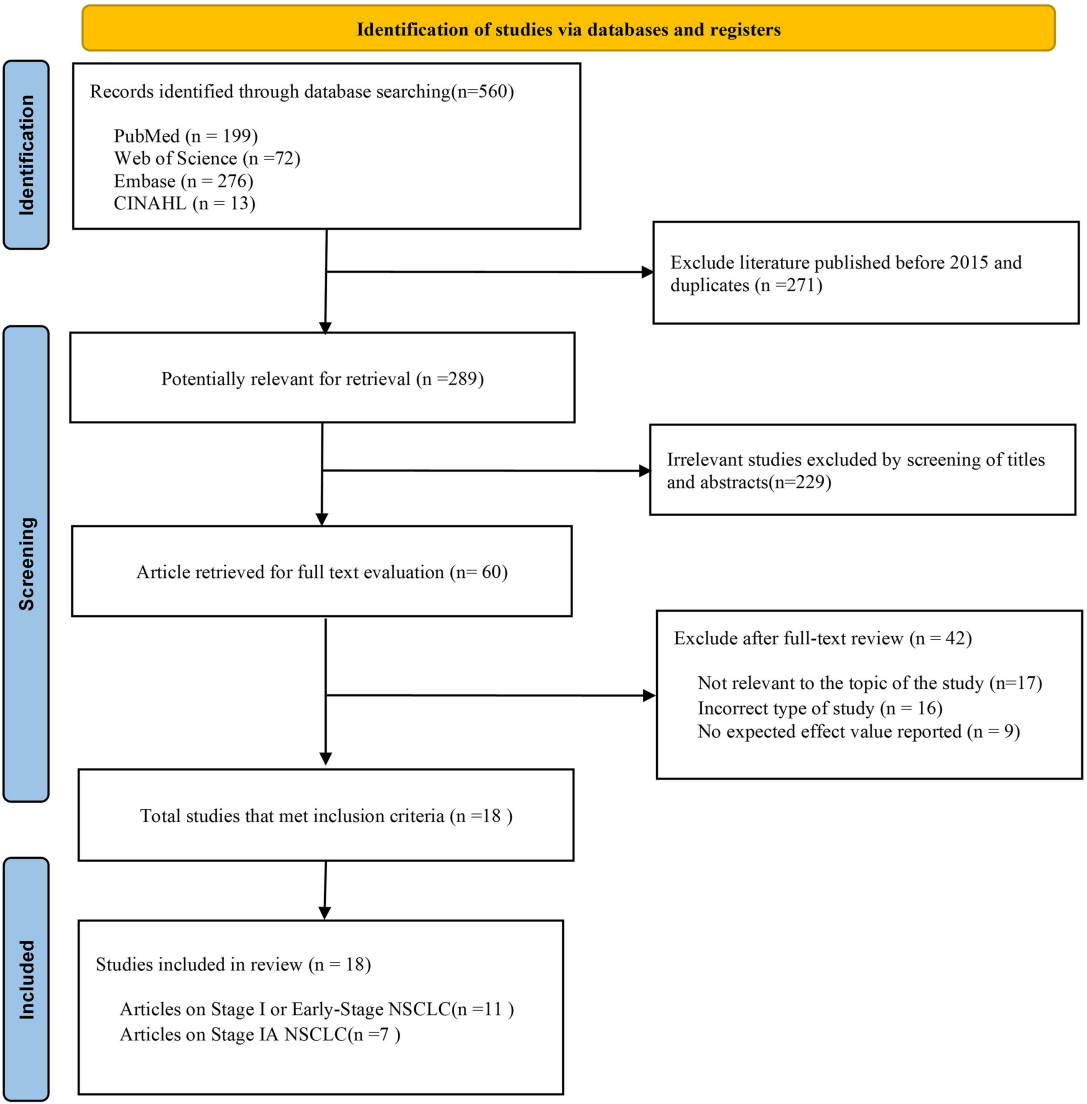
PRISMA flowchart of review search.

As shown in **Figure 2**, a total of 560 records were retrieved from four electronic databases. After removing duplicates and studies published before 2015, 289 records remained for preliminary screening. Following title and abstract screening, 229 irrelevant studies were excluded, leaving 60 articles for full-text assessment. After full-text assessment, 42 articles were excluded for the following reasons: not relevant to the research topic (n = 17), incorrect study type (n = 16), or absence of expected effect estimates (n = 9).

Ultimately, 18 systematic reviews (12, 17–33) or meta-analyses met the inclusion criteria and were included in this umbrella review, as summarized in **Table 1**. These reviews primarily focused on two patient populations: Stage I or early-stage NSCLC (n = 11) and specifically Stage IA NSCLC (n = 7). Across these reviews, the included primary studies were predominantly cohort studies or randomized controlled trials, with the number of primary studies per meta-analysis ranging from 7 to 42.

**Table 1:** General characteristics of systematic reviews and meta-analysis included in the umbrella review.

First author, year	Type	Country region	Intervention comparison	Study design of primary articles	Number of primary articles	Major assessed outcome	Effect size metric	Quality assessment scale	AMSTAR score
Bedetti et al., 2017 (27)	A	Italy	Lobectomy Versus Segmentectomy	Cohort study and RCT	27	OS	HR	Cochrane Tool	High
Bertolaccini et al., 2022 (23)	B	Italy	Lobectomy Versus Segmentectomy	Cohort study	10	OS, DFS	HR	Cochrane RoB Tool	High
Bertolaccini et al., 2024 (28)	B	Italy	Lobectomy Versus Segmentectomy	Cohort study and RCT	40	OS, DFS	HR	NOS + Cochrane RoB + GRADE	High
Fatima et al., 2024 (25)	A	Pakistan	Lobectomy Versus Sublobar resection	Cohort study and RCT	27	OS, DFS	HR	NOS + Cochrane RoB + GRADE	Moderate
Feng et al., 2021 (31)	A	China	Lobectomy Versus Segmentectomy	Cohort study	7	Lung cancer-specific survival and OS	OR	NOS	Critically Low
Fong et al., 2023 (29)	B	Singapore	Lobectomy Versus Sublobar resection	Cohort study	7	OS, DFS	HR	NR	High
Guo et al., 2019 (20)	B	China	Lobectomy Versus Sublobar resection	Cohort study	10	OS and RFS	HR	NR	Moderate
Li et al., 2024 (32)	A	China	Lobectomy Versus Segmentectomy	Cohort study and RCT	17	OS, DFS and RFS	HR	NOS	High
Lin et al., 2024 (19)	B	China	Lobectomy Versus Sublobar resection	Cohort study and RCT	26	OS and RFS	HR	NOS, Cochrane	High
Liu et al.,2016 (17)	A	China	Lobectomy Versus Sublobar resection	NR	8	OS, DFS	HR	NR	Critically Low
Lv et al., 2021 (21)	A	China	Lobectomy Versus Sublobar resection	Cohort study and RCT	12	OS, DFS	HR	NOS	Critically Low
Mamede et al., 2024 (30)	B	Brazil	Lobectomy Versus Sublobar resection	Cohort study and RCT	30	OS, DFS	HR	Cochrane	High
Righi et al., 2023 (24)	B	Italy	Lobectomy Versus Segmentectomy	Cohort study and RCT	14	OS, DFS	HR	NOS	Moderate
Winckelmans et al., 2020 (26)	A	Belgium	Lobectomy Versus Segmentectomy	Cohort study	28	OS, CSS and RFS	HR	Cochrane	Moderate
Zeng et al., 2020 (33)	A	China	Lobectomy Versus Segmentectomy	Cohort study	12	OS, DFS	HR	NOS	High
Zhang 2015 (ATS) (18)	A	China	Lobectomy Versus Segmentectomy	Cohort study	31	RFS and OS	HR	NR	Critically Low
Zhang 2015 (JSO) (22)	A	China	Lobectomy Versus Sublobar resection	NR	42	OS, CSS and RFS	HR	NR	Critically Low
Zheng et al., 2020 (12)	B	China	Lobectomy Versus Segmentectomy	Cohort study	12	OS, DFS	HR	GRADE	High

Type A, mainly studying the outcomes of Stage I or Early-Stage NSCLC; Type B, mainly studying the outcomes of Stage IA NSCLC; Cohort studies include prospective and retrospective designs, regardless of propensity score matching (PSM) application.

OS, overall survival; CSS, cancer-specific survival; RFS, recurrence-free survival; DFS, disease-free survival; HR, hazard ratio; OR, odds ratio; NR, not reported.

### 3.3 Quality assessment

The methodological quality of the 18 included systematic reviews and meta-analyses was critically appraised using the AMSTAR-2 tool. As detailed in **Table 1**, the assessments demonstrated a broad spectrum of methodological quality. Nine reviews were rated as high quality (12 19 23 27–30 32 33), four as moderate quality (20 24–26), and five as critically low quality (17 18 21 22 31).No reviews were rated as “low” quality, indicating a polarization in methodological rigor, with reviews either adhering well to standards or exhibiting critical weaknesses. Furthermore, most studies demonstrated high methodological quality.

The strength of evidence for the primary outcomes was evaluated for each meta-analysis using the GRADE approach, with results presented in **Table 2** (OS) and **Table 3** (DFS). Notably, for comparisons of OS between sublobectomy and lobectomy, most meta-analyses (10 of 18) were graded as providing low or very low certainty evidence, with only one meta-analysis providing moderate-certainty evidence. A similar pattern was observed for DFS, with the evidence predominantly graded as low or very low certainty.

**Table 2:** Methodological quality and detailed results of systematic reviews and meta-analyses comparing overall survival between sublobectomy and lobectomy.

Type	AMSTAR-2	First author, year	No. of primary studies (participants)	Metrics	Original effects estimate (95% CI)	Random-effects estimate (95% CI)	Random-effects P	I²%	95% Prediction interval	Egger’s test P	Largest study estimate (95% CI)	Grade
A	High	Bedetti et al., 2017 (27)	27 (24,542)	HR	1.04 (0.92,1.18)	1.04(0.92,1.18)	0.50	0%	(0.92,1.18)	0.88	0.85 (0.58,1.24)	Moderate
	Moderate	Fatima et al., 2024 (25)	27 (10,449)	HR	1.10 (0.94,1.30)	1.10 (0.94,1.30)	0.23	26%	(0.72,1.68)	0.75	1.05 (0.80,1.35)	Very Low
	Critically Low	Feng et al., 2021 (31)	7 (19,446)	OR	0.86 (0.60,1.23)	0.86 (0.60,1.23)	0.40	91%	(0.37,2.01)	0.89	0.52 (0.45,0.60)	Very Low
	High	Li et al., 2024 (32)	17 (4,476)	HR	1.14 (0.97,1.32)	1.14 (0.97,1.32)	0.10	18%	(0.70,1.85)	0.42	1.16 (0.89,1.52)	Moderate
	Critically Low	Liu et al.,2016 (17)	8 (1,613)	HR	1.45 (1.11,1.90)	1.47(1.02, 2.13)	0.040	27%	(0.80,2.70)	0.92	2.12 (1.38,3.26); 0.95 (0.62,1.46)	Low
	Critically Low	Lv et al., 2021 (21)	12 (4,373)	HR	1.49 (1.05, 2.13)	1.49 (1.05, 2.13)	0.025	76.2%	(0.26,1.73)	0.300	0.95 (0.74,1.22)	Very Low
	Moderate	Winckelmans et al., 2020 (26)	28 (NR)	HR	1.31 (1.10,1.69)	1.31(1.10,1.69)	0.04	0%	(1.07,1.53)	< 0.05	0.49 (0.24,1.00)	Very Low
	High	Zeng et al., 2020 (33)	12 (2,313)	HR	1.11 (0.89,1.38)	1.11 (0.89,1.38)	0.374	0%	(0.89,1.38)	0.644	1.17 (0.89,1.53); 1.19 (0.03,44.79)	Low
	Critically Low	Zhang 2015 (ATS) (18)	31 (27,421)	HR	1.26 (1.14,1.39)	1.26 (1.14,1.39)	< 0.001	25.7%	(1.01,1.59)	0.96	1.37 (1.18,1.59)	Very Low
	Critically Low	Zhang 2015 (JSO) (22)	42 (21,926)	HR	1.53 (1.40,1.67)	1.53 (1.40,1.67)	0.088	32.2%	(0.90,2.60)	0.17	0.95 (0.61,1.47)	Very Low
B	High	Bertolaccini et al., 2022 (23)	10 (1,953)	HR	0.80 (0.49,1.30)	0.85 (0.52,1.38)	0.51	0%	(0.52,1.38)	0.24	0.60 (0.29,1.25)	Moderate
	High	Bertolaccini et al., 2024 (28)	40 (103,926)	HR	1.10 (0.94,1.30)	1.10 (0.94,1.30)	0.24	84%	(0.65,1.87)	0.69	1.72 (1.59,1.86)	Moderate
	High	Fong et al., 2023 (29)	7 (2,528)	HR	0.92 (0.77,1.11)	0.89(0.72,1.09)	0.38	7.5%	(0.68,1.15)	0.28	0.95 (0.72,1.26)	Moderate
	Moderate	Guo et al., 2019 (20)	10 (2,265)	HR	0.96 (0.75,1.24)	0.96 (0.75,1.24)	0.69	37.3%	(0.54,1.72)	0.41	0.94 (0.83,1.05)	Moderate
	High	Lin et al., 2024 (19)	26 (12,667)	HR	1.28 (0.98,1.69)	1.28 (0.98,1.69)	0.07	73%	(0.64,2.57)	0.33	0.66 (0.47,0.93)	Moderate
	High	Mamede et al., 2024 (30)	30 (60,655)	HR	1.27 (1.10,1.47)	1.27 (1.10,1.47)	0.001	69%	(0.92,1.74)	0.21	1.61 (1.52,1.71)	Very Low
	Moderate	Righi et al., 2023 (24)	14 (5,352)	HR	1.05 (0.91,1.21)	0.99 (0.76,1.28)	0.09	38%	(0.52,1.86)	0.44	1.20 (0.99,1.44)	Moderate
	High	Zheng et al., 2020 (12)	12 (8,072)	HR	1.45 (1.23,1.67)	1.45 (1.23,1.67)	< 0.001	0%	(1.18,1.79)	0.251	1.63 (1.38, 1.92)	Low

Type A, systematic reviews mainly including studies focused on Stage I or Early-Stage NSCLC.

Type B, systematic reviews mainly including studies focused on Stage IA NSCLC.

Grading of evidence quality was assessed using a modified approach based on the GRADE framework, considering factors such as risk of bias, inconsistency, indirectness, imprecision, and publication bias.

The random-effects model estimate with its 95% CI is presented as the primary summary measure. The 95% prediction interval indicates the range within which the true effect size of a future study is expected to fall.

Egger’s test was used to assess potential publication bias (P< 0.05 indicates significant bias).

The largest study estimate represents the point estimate from the single largest primary study included in each respective meta-analysis. AMSTAR-2, A Measurement Tool to Assess Systematic Reviews-2; HR, hazard ratio; CI, confidence interval.

**Table 3:** Methodological quality and detailed results of systematic reviews and meta-analyses comparing disease-free survival between sublobectomy and lobectomy.

Type	AMSTAR-2	First author, year	No. of studies (participants)	Metrics	Original effects estimate (95% CI)	Random-effects estimate (95% CI)	Random- effects P	I²%	95% Prediction interval	Egger’s test P	Largest study estimate (95% CI)	Grade
A	Moderate	Fatima et al., 2024 (25)	27(10,449)	HR	1.10 (0.94, 1.29)	1.10 (0.94, 1.29)	0.23	17%	(0.81, 1.50)	0.62	1.01 (0.83, 1.23)	Very Low
	High	Li et al., 2024 (32)	17(4, 476)	HR	1.13 (0.91, 1.41)	1.12 (0.89, 1.42)	0.27	0%	(0.73, 1.70)	0.56	1.10 (0.87, 1.40)	Moderate
	Critically low	Liu et al.,2016 (17)	8(1,613)	HR	1.19 (0.67, 2.10)	1.19 (0.67, 2.10)	0.56	0%	(0.67, 2.10)	0.82	1.13 (0.44, 2.89)	Low
	Critically low	Lv et al., 2021 (21)	12(4,373 )	HR	1.07 (0.88, 1.29)	1.07 (0.88, 1.29)	0.50	0%	(0.88, 1.29)	0.78	0.96 (0.69, 1.31)	Very Low
	High	Zeng et al., 2020 (33)	12 (2,313)	HR	1.09 (0.89, 1.33)	1.09 (0.89, 1.33)	0.42	0%	(0.89, 1.32)	0.43	1.10 (0.87, 1.40); 0.95 (0.61, 1.47)	Very low
B	High	Bertolaccini et al., 2022 (23)	10 (1,953)	HR	1.07 (0.73, 1.56)	1.07 (0.73, 1.56)	0.72	0%	(0.73, 1.56)	0.002	0.95 (0.61, 1.47)	Moderate
	High	Bertolaccini et al., 2024 (28)	40 (103,926)	HR	1.13 (0.95, 1.31)	1.13 (0.95, 1.31)	0.18	0%	(0.85, 1.45)	0.41	1.10 (0.77, 1.49)	Moderate
	High	Fong et al., 2023 (29)	7 (2,528)	HR	1.06 (0.90, 1.24)	1.03 (0.88, 1.21)	0.73	0%	(0.88, 1.21)	0.69	1.01 (0.83, 1.24)	Moderate
	High	Mamede et al., 2024 (30)	30 (60,655)	HR	1.31 (1.08, 1.60)	1.31 (1.08, 1.60)	0.006	57%	(0.95, 1.81)	0.29	1.89 (1.13, 3.17)	Very low
	Moderate	Righi et al., 2023 (24)	14 (5,352)	HR	1.00 (0.83, 1.21)	1.00 (0.78, 1.27)	0.29	18%	(0.61, 1.64)	0.70	1.00 (0.75, 1.32)	Moderate
	High	Zheng et al., 2020 (12)	12 (8,072)	HR	1.03 (0.65, 1.82)	1.03 (0.65, 1.82)	0.76	0%	(0.58,1.85)	0.66	0.83 (0.66, 1.03)	Low

Type A, systematic reviews mainly including studies focused on Stage I or Early-Stage NSCLC.

Type B, systematic reviews mainly including studies focused on Stage IA NSCLC.

Grading of evidence quality was assessed using a modified approach based on the GRADE framework.

The random-effects model estimate with its 95% CI is presented as the primary summary measure.

Egger’s test was used to assess potential publication bias (P< 0.05 indicates significant bias).

The largest study estimate represents the point estimate from the single largest primary study included in each respective meta-analysis. AMSTAR-2, A Measurement Tool to Assess Systematic Reviews-2; HR, hazard ratio; CI, confidence interval.

It is noteworthy that several meta-analyses with high or moderate AMSTAR-2 ratings still produced evidence graded as low or very low certainty under the GRADE framework. This underscores that even well-conducted systematic reviews may synthesize primary evidence with inherent limitations, ultimately resulting in low confidence in effect estimates.

### 3.4 Overlapping and non-overlapping reviews

We further assessed overlap among the included reviews by constructing a citation matrix and calculating the corrected covered area (CCA). Two reviews showed very high overlap in primary studies and were therefore examined in detail (22 27). Because the overlapping reviews differed in methodological quality, recency, and specificity of surgical comparison, Zhang et al., 2015 (J Surg Oncol), which included broader and less specific limited-resection comparisons, was excluded from the updated pooled analysis, whereas Bedetti et al., 2017, a more focused and methodologically stronger review, was retained (22 27). After this exclusion, the overall overlap was reduced to a minor level, with the CCA decreasing to 3.0%. The detailed overlap assessment and rationale for retaining or excluding each review are presented in **Supplementary Table 7**.

### 3.5 Survival outcomes

In this umbrella review, we synthesized evidence from multiple meta-analyses comparing survival outcomes between sublobectomy and lobectomy in patients with Stage I NSCLC (**Tables 2**, **3**).

#### 3.5.1 Overall survival

For overall survival (OS), the pooled results across meta-analyses showed substantial variability. Most meta-analyses reported HRs with confidence intervals crossing 1.00, indicating no statistically significant difference in OS between sublobectomy and lobectomy. For example, high-quality meta-analyses by Bedetti (2017) and Li (2024) reported non-significant HRs of 1.04 (95% CI: 0.92–1.18) and 1.14 (95% CI: 0.97–1.32), respectively (27, 32).

However, several other meta-analyses reached different conclusions. Zhang et al. (2015 JSO), Zhang et al. (2015 ATS) and Mamede (2024) reported statistically significant superiority of lobectomy, with HRs of 1.26 (95% CI: 1.14–1.39), 1.53 (95% CI: 1.40–1.67), and 1.27 (95% CI: 1.10–1.47), respectively (18 22 30). Lv 2021 reported a significant OS difference in the opposite reporting direction; after harmonizing the comparison direction, the estimate corresponded to a higher hazard after sublobectomy. However, this review was rated as critically low quality and showed substantial heterogeneity (21).

#### 3.5.2 Disease-free survival

Regarding disease-free survival (DFS), the evidence was generally more consistent. Most pooled HR estimates clustered around 1.00, with confidence intervals spanning the null value. Representative examples include Fatima (2024) with HR 1.10 (95% CI: 0.94–1.29), Bertolaccini (2024) with HR 1.13 (95% CI: 0.95–1.31), and Fong (2023) with HR 1.03 (95% CI: 0.88–1.21) (25 28 29). A notable exception was Mamede (2024), which reported significantly better DFS with lobectomy (HR 1.31, 95% CI: 1.08–1.60) (30).

#### 3.5.3 Methodological quality influence

Considering methodological quality, the directionally extreme OS findings were largely derived from reviews with important methodological limitations, including Zhang 2015 (ATS), Zhang 2015 (JSO), and Lv 2021, all of which were rated as critically low on AMSTAR-2 (18, 21, 22).

#### 3.5.4 Stage IA subgroup

Based on **Table 2** and **3**, pooled OS results from reviews focusing on Stage IA NSCLC were inconsistent. Most, including the largest by Bertolaccini et al. (2024; n = 103,926) (28), showed no significant OS difference between procedures (HRs 0.85–1.10; 95% CIs crossing 1.0). Two meta-analyses, however, reported significantly worse OS with sublobectomy (12 30): Mamede et al. (2024) (HR = 1.27, 95% CI: 1.10–1.47, P = 0.001) and Zheng et al. (2020) (HR = 1.45, 95% CI: 1.23–1.67, P< 0.001). For DFS, five of six meta-analyses found no significant difference (HRs 1.00–1.13; 95% CIs spanning null). Mamede et al. (2024) again reported significantly worse DFS with sublobectomy (HR = 1.31, 95% CI: 1.08–1.60, P = 0.006). Heterogeneity was generally low across DFS outcomes, except in this review (I² = 57%). Overall, the certainty of DFS evidence ranged from very low to moderate, and interpretation remained limited by heterogeneity and the observational nature of most primary evidence.

### 3.6 Recalculated pooled analyses of survival outcomes

Results of the recalculated pooled analyses are summarized in **Table 4**. In the broader Stage I NSCLC population, OS showed a small but statistically significant higher hazard after sublobectomy (HR 1.09, 95% CI 1.02–1.16; P = 0.011), with substantial heterogeneity (I² = 71.6%) and significant Egger’s test (P< 0.001). DFS also showed a modest higher hazard after sublobectomy (HR 1.13, 95% CI 1.04–1.23; P = 0.005), with low heterogeneity (I² = 9.4%). In Stage IA disease, OS did not differ significantly (HR 1.10, 95% CI 0.99–1.22; P = 0.064), whereas DFS showed a small but statistically significant higher hazard after sublobectomy (HR 1.13, 95% CI 1.01–1.27; P = 0.032). These findings should be interpreted together with the low certainty and heterogeneity of the underlying evidence (10, 16).

**Table 4:** Recalculated pooled analyses of survival outcomes after deduplication.

Analysis	Evidence set	No. of estimates	Random-effects HR (95% CI)	P value	I² (%)	Egger’s test P	Interpretation
Stage I NSCLC OS	All sublobar vs lobectomy	129	1.09 (1.02–1.16)	0.011	71.6	<0.001	Small but statistically significant higher hazard after sublobectomy; exploratory because of high heterogeneity and small-study effects
Stage IA NSCLC OS	All sublobar vs lobectomy	68	1.10 (0.99–1.22)	0.064	77.1	<0.001	Not statistically significant; limited by high heterogeneity and small-study effects
Stage I NSCLC DFS	All sublobar vs lobectomy	63	1.13 (1.04–1.23)	0.005	9.4	0.328	Modest recurrence-related signal with low heterogeneity
Stage IA NSCLC DFS	All sublobar vs lobectomy	45	1.13 (1.01–1.27)	0.032	21.8	0.242	Small but statistically significant recurrence-related signal

The main analyses summarize broad all-sublobar estimates. Because some reviews combined anatomical segmentectomy and wedge resection, surgical-extent classification and sensitivity interpretation are provided in **Supplementary Tables 10**–**12**. HR >1 indicates higher hazard after sublobectomy. NSCLC, non-small cell lung cancer; HR, hazard ratio; CI, confidence interval; I², heterogeneity index, overall survival (OS), disease-free survival (DFS).

### 3.7 Surgical-extent sensitivity synthesis

Because several reviews combined segmentectomy and wedge resection, we performed a surgical-extent sensitivity synthesis (19, 22, 25, 30, 36). In review-level exploratory analyses, segmentectomy-only Stage IA/≤2 cm OS did not differ significantly from lobectomy (HR 1.06, 95% CI 0.94–1.19), and segmentectomy-only Stage IA/≤2 cm DFS/RFS was also not statistically significant (HR 1.07, 95% CI 0.99–1.15). In contrast, broader segmentectomy-only analyses across Stage I or early-stage populations showed modestly higher hazards. These findings suggest that tumor stage, tumor size, and selection criteria remain important sources of heterogeneity. Mixed or wedge-containing sublobar estimates should not be interpreted as direct evidence for anatomical segmentectomy. The surgical-extent classification and exploratory sensitivity analyses are summarized in **Supplementary Tables 10**, **12**.

### 3.8 Summary findings

This umbrella review synthesized evidence from 18 systematic reviews and meta-analyses comparing sublobectomy and lobectomy for Stage I NSCLC. Methodological quality among the included reviews was polarized. Nine were rated as high quality, while the remainder ranged from moderate to critically low. The certainty of evidence for both primary outcomes, overall survival (OS) and disease-free survival (DFS), was predominantly low or very low according to the GRADE framework.

Pooled evidence for OS was characterized by substantial heterogeneity (I² > 70%). While the majority of meta-analyses reported no statistically significant difference between the two procedures, conflicting results were observed in several reviews, favoring either lobectomy or sublobectomy. Evidence for DFS was more consistent than OS, with lower heterogeneity across updated pooled analyses. Although many individual meta-analyses reported non-significant DFS differences, the updated pooled analyses suggested a modestly higher recurrence-related hazard after sublobectomy in both Stage I and Stage IA disease.

In the Stage IA NSCLC subgroup, OS showed no statistically significant difference despite considerable heterogeneity (I² = 77.1%). DFS in this subgroup demonstrated a statistically significant hazard ratio (HR) of 1.13 with low heterogeneity. Analyses focused specifically on Stage IA patients, alongside secondary outcome meta-analyses, confirmed the general pattern of non-significant OS differences accompanied by high heterogeneity.

## 4 Discussion

In this umbrella review, we synthesized 18 meta-analyses and an updated pooled analysis of primary studies comparing lobectomy with sublobar resection for stage I NSCLC. Across reviews, OS estimates varied widely and were often highly heterogeneous (I² >70%). Most meta-analyses reported no statistically significant OS difference between sublobectomy and lobectomy, but some reached opposing conclusions. In our recalculated pooled analysis, sublobectomy was associated with a slightly higher hazard of death in stage I NSCLC (HR 1.09, 95% CI 1.02–1.16). For stage IA, the estimate was imprecise and not statistically significant (HR 1.10, 95% CI 0.99–1.22). Both pooled analyses showed substantial heterogeneity and signals of small-study effects, which reduces confidence in the OS estimates. Therefore, any OS difference is likely small and may reflect residual confounding, patient selection, and variability in surgical technique.

DFS results were more consistent across studies. Across meta-analyses, DFS hazard ratios were close to 1.0 and heterogeneity was generally low. In the updated pooled analysis, sublobectomy was associated with a modestly higher recurrence risk in stage I (HR 1.13, 95% CI 1.04–1.23; I² = 9.4%) and stage IA disease (HR 1.13, 95% CI 1.01–1.27; I² = 21.8%). Heterogeneity was low, and we found no strong evidence of publication bias in these DFS analyses. Although recurrence risk may be slightly higher after sublobectomy, the effect size appears modest. This DFS signal did not consistently translate into worse OS, especially in stage IA disease. Clinically, decisions should balance a small recurrence signal against the functional benefits of parenchymal preservation, particularly in patients with limited pulmonary reserve or substantial comorbidity.

To address this balance more explicitly, we added a supplementary summary of pulmonary function outcomes reported or discussed in the included reviews. Pulmonary function outcomes were not uniformly reported across the included evidence base, and only one included review explicitly evaluated both survival and pulmonary function (30). Therefore, these data were summarized descriptively rather than pooled quantitatively. The available evidence supports the biological and clinical rationale that parenchymal-sparing resection may better preserve postoperative respiratory function, but this functional advantage should be interpreted together with the modest DFS signal favoring lobectomy (6 30).

**Figure 1** illustrates the evolving keyword trends in surgical research on stage I NSCLC. Early research primarily focused on radiation therapy, video-assisted thoracic surgery (VATS), and stereotactic radiotherapy. Subsequently, research attention shifted toward lymph node evaluation, mortality, and evidence synthesis. In recent years, keywords such as overall survival, disease-free survival, segmentectomy, and robotic-assisted surgery have become increasingly prominent, indicating a temporal shift in research focus. The co-occurrence network further demonstrates that lobectomy and segmentectomy remain central research themes, suggesting that the comparative effectiveness of these two approaches continues to be a core focus. Overall, the field has shifted toward more precise and minimally invasive surgical strategies. However, the AMSTAR-2 and GRADE assessments indicate that increased publication activity has not consistently translated into high-certainty comparative evidence.

These findings should be interpreted in the context of recent advances in precision thoracic surgery and pivotal randomized evidence. The JCOG0802/WJOG4607L trial reported favorable overall survival with segmentectomy despite a higher local recurrence rate, whereas CALGB 140503 demonstrated non-inferiority of sublobar resection for carefully selected peripheral T1aN0 tumors (34 35). In parallel, improvements in high-resolution CT-based tumor characterization, minimally invasive surgery, robotic-assisted approaches, anatomic segmentectomy, surgical margin planning, and lymph node assessment may have narrowed the prognostic gap between lobectomy and sublobectomy in contemporary practice (23 28 30 34 35). This interpretation is also consistent with our bibliometric findings, in which pulmonary segmentectomy, multicenter studies, and robotic-assisted thoracoscopic surgery emerged as recent research hotspots. These randomized trials provide the highest-level evidence for carefully selected small peripheral tumors (34, 35). However, the broader meta-analytic literature remains heterogeneous because many reviews combine historical cohorts, registry-based studies, compromised resections, intentional resections, segmentectomy, and wedge resection (19, 22, 25, 30, 36). Therefore, the present umbrella review is best interpreted as an evidence-quality and applicability assessment of the existing secondary literature rather than as a replacement for randomized evidence (10, 11, 16).

The substantial heterogeneity observed in OS analyses underscores that sublobectomy should not be considered a uniform entity. Differences between anatomical segmentectomy and wedge resection, tumor size and consolidation ratio, surgical margin adequacy, lymph node sampling, and biological features such as spread through air spaces (STAS) likely contribute to outcome variability (19 22 36). Future research should incorporate stratified analyses and individual patient data approaches to refine selection criteria.

Another important source of heterogeneity may be the evolution of TNM staging definitions during the study period. The included literature spans years in which both the 7th and 8th editions of the TNM classification were used. Compared with the 7th edition, the 8th edition introduced more refined T1 subcategories and placed greater emphasis on tumor size and the invasive or solid component (4 37). Therefore, patients labeled as Stage IA in earlier and later studies may not be fully comparable. Because this umbrella review was based on aggregate data from published systematic reviews and meta-analyses, individual patient-level restaging according to a unified TNM edition was not feasible. Nevertheless, staging evolution should be considered an important contributor to the high OS heterogeneity, particularly in the Stage IA subgroup (4 37).

## 5 Limitations

This umbrella review has several limitations. Most primary studies were observational, and residual confounding or selection bias could not be fully excluded. Although overlap was assessed and duplicated primary-study entries were removed, residual overlap may remain, especially among registry-based studies (11). Sublobar resection was also inconsistently defined, with some reviews evaluating segmentectomy alone and others combining segmentectomy with wedge resection (19, 22, 25, 30, 36). In addition, the surgical-extent and publication-bias analyses were based on review-level aggregate estimates and should be interpreted as exploratory. Differences in TNM editions and incomplete reporting of functional and pathological variables further limited subgroup analyses (37).

## 6 Conclusion

This umbrella review suggests that the comparative effectiveness of lobectomy and sublobar resection in Stage I NSCLC depends on patient selection, tumor characteristics, and the type of sublobar resection performed (19, 22, 25, 30, 36). In updated all-sublobar pooled analyses, sublobar resection was associated with small but statistically significant higher hazards for OS and DFS in the broader Stage I population, whereas Stage IA OS did not differ significantly. Because OS estimates were limited by heterogeneity, small-study effects, overlap, and low certainty, these findings support individualized, risk-adapted use of parenchymal-sparing surgery rather than uniform replacement of lobectomy (10, 11, 16, 34, 35).

## Data Availability

Publicly available datasets were analyzed in this study. This data can be found here: https://webofscience.clarivate.cn/wos/alldb/basic-search and https://pubmed.ncbi.nlm.nih.gov/.
