# Supplementary material for: Lobectomy versus sublobar resection for stage I non-small cell lung cancer: an umbrella review of evidence quality, overlap, surgical-extent heterogeneity, and survival outcomes
Source: Front Oncol. 2026 Jun 5;16:1836910. doi: 10.3389/fonc.2026.1836910 (PMC13279088; doi:10.3389/fonc.2026.1836910)
Supplement: Supplementary file 1 [file Table1.docx]

Table of content

Table S1. Search strategy in Web of Science Core Collection (updated Sep 2025)

Table S2. Search strategy for systematic review or meta-analysis (updated on Sept 2025)

Table S3. Inclusion and exclusion criteria

Table S4. AMSTAR-2 Assessment

Table S5. GRADE certainty of evidence for the 18 included meta-analyses

Table S6. Summary of data extraction domains

Table S7. Overlap and methodological comparison between Bedetti et al. (2017) and Zhang et al. (2015, J Surg Oncol)

Table S8. PRISMA_2020_checklist

Table S9. Summary of pulmonary function outcomes reported or discussed in included reviews

Table S10. Surgical-extent classification of included reviews

Table S11. Deduplication and overlap adjudication for updated pooled analyses

Table S12. Review-level exploratory sensitivity analyses and publication-bias interpretation

Table S1. Search strategy in Web of Science Core Collection (updated Sep 2025)

| Keywords | Search terms |
| --- | --- |
| Lung cancer; Stage I NSCLC; Lobectomy; Segmentectomy; Sublobar resection; Limited resection; Meta-analysis | #1. TS = ( "lung cancer" OR "pulmonary carcinoma" OR "non-small cell lung cancer" OR NSCLC OR bronchogenic )#2. TS = ( "stage I" OR "stage IA" OR "stage IB" OR "early-stage" OR "early stage" OR T1 OR T1a OR T1b )#3. TS = ( lobectom* )#4. TS = ( sublobar* OR segmentectom* OR "anatomic segmentectom*" OR "wedge resect*" OR "limited resection" )#5. NOT TS = ( mouse OR mice OR rat OR murine OR animal OR canine )#6. Timespan = 2015–2025Final search = #1 AND #2 AND #3 AND #4 AND #6 AND NOT #5 |

Table S2. Search strategy for systematic review or meta-analysis (updated on Sept 2025)

| Database | Keywords | Search terms | |
| --- | --- | --- | --- |
| Web of Science | Lung cancer; Stage I; Surgical procedures; Sublobar resection; Meta-analysis | | #1 = TS=("lung cancer" OR "pulmonary carcinoma" OR "non-small cell lung cancer" OR NSCLC OR bronchogenic) #2 = TS=("stage I" OR "stage IA" OR "stage IB" OR "early-stage" OR "early stage" OR T1 OR T1a OR T1b) #3 = TS=(lobectom*) #4 = TS=(sublobar* OR segmentectom* OR "anatomic segmentectom*" OR "wedge resect*" OR "limited resection") #5 = NOT TS=(mouse OR mice OR rat OR murine OR animal OR canine) #6 = TS=(meta) Final search = #1 AND #2 AND #3 AND #4 AND #6 |
| PubMed | Lung cancer; Stage I; Lobectomy; Segmentectomy; Meta-analysis | #1 = ("lung cancer"[Title/Abstract] OR "pulmonary carcinoma"[Title/Abstract] OR "non-small cell lung cancer"[Title/Abstract] OR NSCLC[Title/Abstract]) #2 = ("stage I"[Title/Abstract] OR "stage IA"[Title/Abstract] OR "stage IB"[Title/Abstract] OR "early-stage"[Title/Abstract] OR "early stage"[Title/Abstract]) #3 = (lobectomy[Title/Abstract]) #4 = (sublobar[Title/Abstract] OR segmentectomy[Title/Abstract] OR "wedge resection"[Title/Abstract] OR "limited resection"[Title/Abstract]) #5 = (Review[pt] OR Meta-Analysis[pt]) Final search = #1 AND #2 AND #3 AND #4 AND #5 | |
| Embase | NSCLC; Stage I; Sublobar resection; Randomized / meta evidence | #1 = ('non small cell lung cancer'/exp OR 'non small cell lung cancer':ti,ab OR 'lung cancer':ti,ab OR nsclc:ti,ab) #2 = ('stage i':ti,ab OR 'stage ia':ti,ab OR 'stage ib':ti,ab OR 'early stage':ti,ab OR 'early-stage':ti,ab) #3 = ('lobectomy'/exp OR lobectomy:ti,ab) #4 = ('sublobar resection':ti,ab OR 'segmentectomy'/exp OR segmentectomy:ti,ab OR 'wedge resection':ti,ab OR 'limited resection':ti,ab) #5 = ('meta analysis'/exp OR 'systematic review'/exp OR meta:ti,ab OR "systematic review":ti,ab) Final search = #1 AND #2 AND #3 AND #4 AND #5 | |
| CINAHL | Lung cancer; Early-stage NSCLC; Lobectomy; Sublobar procedures; Systematic review | ((MH "Lung Neoplasms+") OR "lung cancer" OR "pulmonary carcinoma" OR "non-small cell lung cancer" OR NSCLC) AND ("stage I" OR "stage IA" OR "stage IB" OR "early-stage" OR "early stage") AND ((MH "Lobectomy") OR lobectomy) AND ("sublobar resection" OR (MH "Segmentectomy") OR segmentectomy OR "wedge resection" OR "limited resection") AND (MH "Meta Analysis" OR MH "Systematic Review") | |
| Cochrane Library | Lung cancer; Stage I; Lobectomy; Segmentectomy | ("lung cancer" OR "pulmonary carcinoma" OR "non-small cell lung cancer" OR NSCLC) AND ("stage I" OR "stage IA" OR "stage IB" OR "early-stage" OR "early stage") AND (lobectomy) AND ("sublobar resection" OR segmentectomy OR "wedge resection" OR "limited resection") AND ("systematic review" OR "meta-analysis") | |

Table S3. Inclusion and exclusion criteria

| Inclusion Criteria | Exclusion Criteria |
| --- | --- |
| 1. Adult patients (≥18 years) with pathologically confirmed stage I NSCLC (IA–IB). | 1. Studies without explicit stage I subgroup data, or where stage I data cannot be separated. |
| 2. Meta-analyses or systematic reviews comparing lobectomy versus sublobectomy (segmentectomy or wedge resection). | 2. Narrative reviews, expert opinions, editorials, comments, letters. |
| 3. Reported primary outcomes: overall survival (OS): major outcome, disease-free survival (DFS), cancer-specific survival (CSS), recurrence rates (local/regional/distant). | 3. Single primary studies (RCTs or cohorts) without synthesis. |
| 4. Reported secondary outcomes: postoperative complications, 30-/90-day mortality, operative time, blood loss, length of hospital stays. | 4. Studies lacking extractable HR/RR/OR or insufficient data for pooled effect estimates. |
| 5. Systematic reviews/meta-analyses including RCTs and/or observational studies. | 5. Animal studies, molecular biology studies, imaging-only research. |
| 6. Mixed-stage studies included only if stage I (stage IA) data were extractable. | 6. Duplicate publications or overlapping datasets (the most complete version retained). |
| 7. Publications written in English. | 7. Non-English publications. |

Table S4. AMSTAR-2 Assessment

| Study | Item 1 | Item 2 | Item 3 | Item 4 | Item 5 | Item 6 | Item 7 | Item 8 | Item 9 | Item 10 | Item 11 | Item 12 | Item 13 | Item 14 | Item 15 | Item 16 | Overall Rating |
| --- | --- | --- | --- | --- | --- | --- | --- | --- | --- | --- | --- | --- | --- | --- | --- | --- | --- |
| Bedetti 2017 | Yes | Yes | Yes | Yes | Yes | Yes | Yes | Yes | Yes | No | Yes | Yes | Yes | Yes | Yes | Yes | High |
| Bertolaccini 2022 | Yes | Yes | Yes | Yes | Yes | Yes | Yes | Yes | Yes | No | Yes | Yes | Yes | Yes | Yes | Yes | High |
| Bertolaccini 2024 | Yes | Yes | Yes | Yes | Yes | Yes | Yes | Yes | Yes | No | Yes | Yes | Yes | Yes | Yes | Yes | High |
| Fatima 2024 | Yes | No | Yes | Yes | Yes | Yes | Yes | Partial Yes | Yes | No | Yes | Yes | No | Yes | Yes | Yes | Moderate |
| Feng 2021 | Yes | No | Yes | Yes | Yes | Partial Yes | Yes | No | No | No | Yes | No | No | Yes | No | Yes | Critically low |
| Fong 2023 | Yes | Yes | Yes | Yes | Yes | Yes | Yes | Yes | Yes | No | Yes | Yes | Yes | Yes | Yes | Yes | High |
| Guo 2019 | Yes | No | Yes | Yes | Yes | Yes | Yes | Partial Yes | No | No | Yes | No | No | Yes | Yes | Yes | Moderate |
| Li 2024 | Yes | Yes | Yes | Yes | Yes | Yes | Yes | Yes | Yes | No | Yes | Yes | Yes | Yes | Yes | Yes | High |
| Lin 2024 | Yes | No | Yes | Yes | Yes | Yes | Yes | Yes | Yes | No | Yes | Yes | Yes | Yes | Yes | Yes | High |
| Liu 2016 | Yes | No | Yes | Partial Yes | Partial Yes | Partial Yes | Yes | No | No | No | Yes | No | No | Yes | No | Yes | Critically low |
| Lv 2021 | Yes | No | Yes | Yes | Yes | Partial Yes | Yes | No | No | No | Yes | No | No | Yes | No | Yes | Critically low |
| Mamede 2024 | Yes | Yes | Yes | Yes | Yes | Yes | Yes | Yes | Yes | No | Yes | Yes | Yes | Yes | Yes | Yes | High |
| Righi 2023 | Yes | No | Yes | Yes | Yes | Yes | Yes | Partial Yes | No | No | Yes | No | No | Yes | Yes | Yes | Moderate |
| Winckelmans 2020 | Yes | No | Yes | Yes | Yes | Yes | Yes | Partial Yes | Yes | No | Yes | No | Yes | Yes | Yes | Yes | Moderate |
| Zeng 2020 | Yes | Yes | Yes | Yes | Yes | Yes | Yes | Yes | Yes | No | Yes | Yes | Yes | Yes | Yes | Yes | High |
| Zhang 2015 (ATS) | Yes | No | Yes | Yes | Yes | Yes | Yes | No | No | No | Yes | No | No | Yes | Partial Yes | Yes | Critically low |
| Zhang 2015 (JSO) | Yes | No | Yes | Yes | Yes | Yes | Yes | No | No | No | Yes | No | No | Yes | Partial Yes | Yes | Critically low |
| Zheng 2020 | Yes | No | Yes | Yes | Yes | Yes | Yes | Yes | Yes | No | Yes | Yes | Yes | Yes | Yes | Yes | High |

Note:

Item 1: Did the research questions and inclusion criteria for the review include the components of PICO?

Item 2: Did the report of the review contain an explicit statement that the review methods were established prior to conduct of the review and did the report justify any significant deviations from the protocol?

Item 3: Did the review authors explain their selection of the study designs for inclusion in the review?

Item 4: Did the review authors use a comprehensive literature search strategy?

Item 5: Did the review authors perform study selection in duplicate?

Item 6: Did the review authors perform data extraction in duplicate?

Item 7: Did the review authors provide a list of excluded studies and justify the exclusions?

Item 8: Did the review authors describe the included studies in adequate detail?

Item 9: Did the review authors use a satisfactory technique for assessing the risk of bias (RoB) in individual studies that were included in the review?

Item 10: Did the review authors report on the sources of funding for the studies included in the review?

Item 11: If meta-analysis was justified did the review authors use appropriate methods for statistical combination of results?

Item 12: If meta-analysis was performed did the review authors assess the potential impact of RoB in individual studies on the results of the meta-analysis or other evidence synthesis?

Item 13: Did the review authors account for RoB in individual studies when interpreting/discussing the results of the review?

Item 14: Did the review authors provide a satisfactory explanation for, and discussion of, any heterogeneity observed in the results of the review?

Item 15: If they performed quantitative synthesis did the review authors carry out an adequate investigation of publication bias (small study bias) and discuss its likely impact on the results of the review?

Item 16: Did the review authors report any potential sources of conflict of interest, including any funding they received for conducting the review?

AMSTAR-2 evaluates methodological rigor across 16 domains. The seven critical domains are Items 2, 4, 7, 9, 11, 13, and 15. Overall ratings were assigned as follows: High, no or one non-critical weakness; Moderate, more than one non-critical weakness but no critical flaw; Low, one critical flaw with or without non-critical weaknesses; Critically low, more than one critical flaw.

Table S5. GRADE certainty of evidence for the 18 included meta-analyses

| Study | Risk of Bias | Inconsistency | Indirectness | Imprecision | Publication Bias | Overall Certainty |
| --- | --- | --- | --- | --- | --- | --- |
| Bedetti 2017 | Serious | No serious inconsistency | No | Some | Low | Moderate |
| Bertolaccini 2022 | Serious | No | No | Some | Low | Moderate |
| Bertolaccini 2024 | Serious | Some | No | Some | Low | Moderate (RCT) / Very Low (Obs) |
| Fatima 2024 | Very serious | Some | No | Serious | Low | Very Low / Moderate (high-risk subgroup) |
| Feng 2021 | Serious | No | No | Serious | Serious | Very Low |
| Fong 2023 | Low | No | No | Some | Low | Moderate–High |
| Guo 2019 | Serious | Some | No | Serious | Some | Low–Moderate |
| Li 2024 | Low | No | No | Some | Low | Moderate |
| Lin 2024 | Low | No | No | Some | Low | Moderate |
| Liu 2016 | Serious | Some | No | Serious | Serious | Low |
| Lv 2021 | Very serious | Serious | No | Serious | Serious | Very Low |
| Mamede 2024 | Serious | Some | No | Some | Low | Very Low / Moderate (RCT-only) |
| Righi 2023 | Low | Some | No | Some | Low | Moderate |
| Winckelmans 2020 | Serious | Serious | No | Serious | Some | Very Low |
| Zeng 2020 | Low | No | No | Some | Low | Low |
| Zhang 2015 (ATS) | Very serious | Serious | No | Some | Serious | Very Low |
| Zhang 2015 (JSO) | Very serious | Serious | No | Some | Serious | Very Low |
| Zheng 2020 | Low | Some | No | Some | Low | Low |

Note：GRADE (Grading of Recommendations, Assessment, Development and Evaluations) evaluates the certainty of evidence across five key domains:

1. Risk of Bias – quality of included studies
2. Inconsistency – heterogeneity (I²), direction of effects
3. Indirectness – applicability of population, intervention, comparator, outcome
4. Imprecision – wide confidence intervals, low event numbers
5. Publication Bias – funnel plot asymmetry, selective reporting

Certainty grading system:

- High: Very confident in the effect estimate
- Moderate: Moderately confident; true effect is likely close
- Low: Limited confidence; true effect may differ substantially
- Very Low: Very little confidence

Observational meta-analyses start at Low and are downgraded further; RCT-based evidence may start at High.

Table S6. Summary of data extraction domains

| Domain | Variables Extracted |
| --- | --- |
| Study characteristics | First author, publication year, country/region, number of included primary studies, study design of primary studies (RCTs / cohorts). |
| Patient characteristics | Total sample size, age range, tumor size subgroups, pathological stage. |
| Intervention and comparator | Type of sublobectomy, definition of lobectomy, surgical approach (VATS vs open), extent of lymph node dissection. |
| Primary outcomes | OS, DFS, CSS, recurrence rates (local, regional, distant), pooled hazard ratios and 95% CI. |
| Secondary outcomes | Postoperative complications, blood loss, operative time, length of hospital stay, 30-day/90-day mortality, OR/RR with 95% CI. |
| Meta-analytic information | Effect model used (fixed/random), heterogeneity (I², Q-test), publication bias (Egger’s test, funnel plot). |
| Quality assessment | AMSTAR-2 item-level ratings; overall quality grade. |
| Evidence grading | GRADE domains and final certainty level. |

Table S7. Overlap and methodological comparison between Bedetti et al. (2017) and Zhang et al. (2015, J Surg Oncol)

| Item | Bedetti et al., 2017 (J Thorac Dis) | Zhang et al., 2015 (J Surg Oncol) | Comment |
| --- | --- | --- | --- |
| Publication year / Journal | 2017, *Journal of Thoracic Disease* | 2015, *Journal of Surgical Oncology* | Bedetti is more recent. |
| Objective | To compare segmentectomy vs lobectomy for stage I NSCLC with OS as primary outcome. | To compare lobectomy vs limited resection (segmentectomy + wedge) and also segmentectomy vs lobectomy as a subgroup. | Zhang includes mixed limited resection, less focused on the specific comparison. |
| Number of included studies | 27 | 42 | Both large meta-analyses. |
| Time window of included primary studies | 1990–2016 | 1980–2014 | Time windows strongly overlap; Zhang includes more early historical cohorts. |
| Target population | Stage I NSCLC (with IA subgroup analyses) | Stage I NSCLC (including IA ≤ 2 cm and older-patient subgroups) | Populations highly comparable. |
| Surgical comparison analyzed | Segmentectomy vs lobectomy only | Lobectomy vs limited resection; segmentectomy vs lobectomy; segmentectomy vs wedge | Bedetti is focused, Zhang includes heterogeneous contrasts. |
| Effect measure for OS | HR (seg vs lob) | HR (multiple contrasts including seg vs lob) | Both report HR, compatible for umbrella synthesis. |
| Main OS result | HR = 1.04 (95% CI 0.92–1.18); no inferiority of segmentectomy | HR = 1.231 (95% CI 1.070–1.417); lobectomy superior | Same comparison but conflicting direction; however the evidence base is overlapping. |
| Stage definition | Stage I (IA/IB) | Stage I with additional subgroup stratification | Similar stage definition. |
| Overlap of primary studies | Shares most classical segmentectomy vs lobectomy cohorts: Ginsberg 1995, Okada 2001, Koike 2003, Yamato 2008, Zhong 2012, Tsutani 2014, etc. | Includes the same historical cohorts (Fan 2012, Liu 2014, Okada 2001, Yamato 2008, Zhong 2012, etc.) | Substantial overlap; evidence not independent. |
| Methodological characteristics | Focused comparison; modern data; low heterogeneity; AMSTAR-2 = High | Broader scope; combines different limited resections; AMSTAR-2 = Critically low | Methodological quality favors Bedetti. |
| Evidence certainty (GRADE) | Moderate to high (consistent direction; no major publication bias) | Low to moderate (indirectness due to mixed limited resection) | Bedetti provides higher-certainty evidence. |
| Evidence independence | Low (shares cohorts with Zhang) | Low (shares cohorts with Bedetti) | Strong redundancy. |
| Overall assessment | High-quality, focused, up-to-date meta-analysis | Broader but less precise for segmentectomy-vs-lobectomy | Bedetti preferred for umbrella meta-analysis. |

Table S8. PRISMA_2020_checklist

| **Section and Topic** | **Item #** | **Checklist item** | **Location where item is reported** |
| --- | --- | --- | --- |
| **TITLE** | | |  |
| Title | 1 | Identify the report as a systematic review. | Title page |
| **ABSTRACT** | | |  |
| Abstract | 2 | See the PRISMA 2020 for Abstracts checklist. | Pages 1-2 |
| **INTRODUCTION** | | |  |
| Rationale | 3 | Describe the rationale for the review in the context of existing knowledge. | Pages 3-4 |
| Objectives | 4 | Provide an explicit statement of the objective(s) or question(s) the review addresses. | Page 4 |
| **METHODS** | | |  |
| Eligibility criteria | 5 | Specify the inclusion and exclusion criteria for the review and how studies were grouped for the syntheses. | Page 5 |
| Information sources | 6 | Specify all databases, registers, websites, organisations, reference lists and other sources searched or consulted to identify studies. Specify the date when each source was last searched or consulted. | Pages 4-5 |
| Search strategy | 7 | Present the full search strategies for all databases, registers and websites, including any filters and limits used. | Pages 4-5 |
| Selection process | 8 | Specify the methods used to decide whether a study met the inclusion criteria of the review, including how many reviewers screened each record and each report retrieved, whether they worked independently, and if applicable, details of automation tools used in the process. | Supplement |
| Data collection process | 9 | Specify the methods used to collect data from reports, including how many reviewers collected data from each report, whether they worked independently, any processes for obtaining or confirming data from study investigators, and if applicable, details of automation tools used in the process. | Supplement |
| Data items | 10a | List and define all outcomes for which data were sought. Specify whether all results that were compatible with each outcome domain in each study were sought (e.g. for all measures, time points, analyses), and if not, the methods used to decide which results to collect. | Page 6 |
|  | 10b | List and define all other variables for which data were sought (e.g. participant and intervention characteristics, funding sources). Describe any assumptions made about any missing or unclear information. | Pages 6-7 |
| Study risk of bias assessment | 11 | Specify the methods used to assess risk of bias in the included studies, including details of the tool(s) used, how many reviewers assessed each study and whether they worked independently, and if applicable, details of automation tools used in the process. | Pages 6-7 |
| Effect measures | 12 | Specify for each outcome the effect measure(s) (e.g. risk ratio, mean difference) used in the synthesis or presentation of results. | Page 8 |
| Synthesis methods | 13a | Describe the processes used to decide which studies were eligible for each synthesis (e.g. tabulating the study intervention characteristics and comparing against the planned groups for each synthesis (item #5)). | Pages 7-9 |
|  | 13b | Describe any methods required to prepare the data for presentation or synthesis, such as handling of missing summary statistics, or data conversions. | Pages 7-9 |
|  | 13c | Describe any methods used to tabulate or visually display results of individual studies and syntheses. | Pages 7-9 |
|  | 13d | Describe any methods used to synthesize results and provide a rationale for the choice(s). If meta-analysis was performed, describe the model(s), method(s) to identify the presence and extent of statistical heterogeneity, and software package(s) used. | Pages 7-9 |
|  | 13e | Describe any methods used to explore possible causes of heterogeneity among study results (e.g. subgroup analysis, meta-regression). | Pages 7-9 |
|  | 13f | Describe any sensitivity analyses conducted to assess robustness of the synthesized results. | Pages 7-9 |
| Reporting bias assessment | 14 | Describe any methods used to assess risk of bias due to missing results in a synthesis (arising from reporting biases). | Pages 7-9 |
| Certainty assessment | 15 | Describe any methods used to assess certainty (or confidence) in the body of evidence for an outcome. | Pages 7-9 |
| **RESULTS** | | |  |
| Study selection | 16a | Describe the results of the search and selection process, from the number of records identified in the search to the number of studies included in the review, ideally using a flow diagram. | Page 11 |
|  | 16b | Cite studies that might appear to meet the inclusion criteria, but which were excluded, and explain why they were excluded. | Pages 11 20 |
| Study characteristics | 17 | Cite each included study and present its characteristics. | Pages 10 |
| Risk of bias in studies | 18 | Present assessments of risk of bias for each included study. | Page 12 |
| Results of individual studies | 19 | For all outcomes, present, for each study: (a) summary statistics for each group (where appropriate) and (b) an effect estimate and its precision (e.g. confidence/credible interval), ideally using structured tables or plots. | Pages 12-13 |
| Results of syntheses | 20a | For each synthesis, briefly summarise the characteristics and risk of bias among contributing studies. | Page 12 |
|  | 20b | Present results of all statistical syntheses conducted. If meta-analysis was done, present for each the summary estimate and its precision (e.g. confidence/credible interval) and measures of statistical heterogeneity. If comparing groups, describe the direction of the effect. | Pages 12–15 |
|  | 20c | Present results of all investigations of possible causes of heterogeneity among study results. | Pages 12–15 |
|  | 20d | Present results of all sensitivity analyses conducted to assess the robustness of the synthesized results. | Pages 12-13, 15 |
| Reporting biases | 21 | Present assessments of risk of bias due to missing results (arising from reporting biases) for each synthesis assessed. | Pages 12-13, 15 |
| Certainty of evidence | 22 | Present assessments of certainty (or confidence) in the body of evidence for each outcome assessed. | Pages 12-13, 15 |
| **DISCUSSION** | | |  |
| Discussion | 23a | Provide a general interpretation of the results in the context of other evidence. | Pages 18-22 |
|  | 23b | Discuss any limitations of the evidence included in the review. | Pages 18-22 |
|  | 23c | Discuss any limitations of the review processes used. | Pages 18-22 |
|  | 23d | Discuss implications of the results for practice, policy, and future research. | Pages 18-22 |
| **OTHER INFORMATION** | | |  |
| Registration and protocol | 24a | Provide registration information for the review, including register name and registration number, or state that the review was not registered. | Page 5 |
|  | 24b | Indicate where the review protocol can be accessed, or state that a protocol was not prepared. | Page5 |
|  | 24c | Describe and explain any amendments to information provided at registration or in the protocol. | Page 5 |
| Support | 25 | Describe sources of financial or non-financial support for the review, and the role of the funders or sponsors in the review. | Pages 23-24 |
| Competing interests | 26 | Declare any competing interests of review authors. | Pages 23-24 |
| Availability of data, code and other materials | 27 | Report which of the following are publicly available and where they can be found: template data collection forms; data extracted from included studies; data used for all analyses; analytic code; any other materials used in the review. | Pages 23-24 |

*From:*  Page MJ, McKenzie JE, Bossuyt PM, Boutron I, Hoffmann TC, Mulrow CD, et al. The PRISMA 2020 statement: an updated guideline for reporting systematic reviews. BMJ 2021;372:n71. doi: 10.1136/bmj.n71. This work is licensed under CC BY 4.0. To view a copy of this license, visit <https://creativecommons.org/licenses/by/4.0/>

Table S9. Summary of pulmonary function outcomes reported or discussed in included reviews

| Study | Surgical comparison | Pulmonary function outcome reported | Extractable pulmonary function metrics | Direction of evidence | Comment |
| --- | --- | --- | --- | --- | --- |
| Bedetti 2017 | Segmentectomy vs lobectomy | Discussed qualitatively | Not pooled as a pulmonary function endpoint | Favored parenchymal preservation conceptually | This review focused mainly on OS. It discussed the parenchyma-sparing rationale of segmentectomy but did not provide a dedicated pooled pulmonary function analysis [27]. |
| Bertolaccini 2022 | Segmentectomy vs lobectomy | Not clearly reported | Not available | Not assessable | The review mainly focused on oncologic outcomes, including OS and DFS. Pulmonary function was not reported as a distinct pooled outcome [23]. |
| Bertolaccini 2024 | Segmentectomy vs lobectomy | Not reported as a dedicated pulmonary function endpoint | Not available | Not assessable | This review included perioperative and survival outcomes, such as OS, DFS, local recurrence, harvested lymph nodes, postoperative morbidity, and length of hospital stay, but pulmonary function was not listed as a primary extracted outcome [28]. |
| Fatima 2024 | Sublobar resection vs lobectomy | Not clearly reported | Not available | Not assessable | The review focused on oncological outcomes, mainly OS and DFS. Pulmonary function data were not sufficiently extractable [25]. |
| Feng 2021 | Segmentectomy vs lobectomy | Not reported | Not available | Not assessable | This review mainly assessed OS and lung cancer-specific survival [31]. |
| Fong 2023 | Sublobar resection vs lobectomy | Not reported | Not available | Not assessable | This patient-level meta-analysis focused on survival and recurrence-related outcomes in small stage IA NSCLC. Pulmonary function was not a main pooled endpoint [29]. |
| Guo 2019 | Sublobar resection vs lobectomy | Not reported | Not available | Not assessable | The review focused on solid-dominant stage IA NSCLC and oncological outcomes [20]. |
| Li 2024 | Segmentectomy vs lobectomy | Not clearly reported | Not available | Not assessable | The review focused on survival outcomes, including OS, DFS, and RFS [32]. |
| Lin 2024 | Segmentectomy/wedge resection vs lobectomy | Not reported | Not available | Not assessable | The review mainly compared oncologic outcomes by sublobar resection type in solid-dominant stage IA disease [19]. |
| Liu 2016 | Sublobectomy vs lobectomy | Not reported | Not available | Not assessable | The review mainly reported OS and DFS [17]. |
| Lv 2021 | Sublobectomy vs lobectomy | Not reported | Not available | Not assessable | The review mainly focused on OS and DFS [21]. |
| Mamede 2024 | Sublobar resection vs lobectomy | Yes | Pulmonary function outcomes were discussed in this review, but extractable standardized pooled FEV1 or DLCO estimates were not consistently available for harmonized synthesis. | Generally favored better functional preservation after sublobar resection | This was the only included review whose title and scope explicitly addressed both survival and pulmonary function in stage IA NSCLC. It is the key source supporting the functional-benefit discussion [30]. |
| Righi 2023 | Segmentectomy vs lobectomy | Not clearly reported | Not available | Not assessable | The review focused on oncologic outcomes for clinical stage I NSCLC up to 2 cm [24]. |
| Winckelmans 2020 | Segmentectomy vs lobectomy | Not reported as a dedicated endpoint | Not available | Not assessable | The review focused mainly on OS, CSS, and RFS [26]. |
| Zeng 2020 | VATS segmentectomy vs VATS lobectomy | Not reported | Not available | Not assessable | The review mainly assessed OS and DFS [33]. |
| Zhang 2015 (ATS) | Segmentectomy vs lobectomy | Not reported as a pooled pulmonary function endpoint | Not available | Not assessable | The review focused mainly on RFS and OS [18]. |
| Zhang 2015 (JSO) | Lobectomy vs segmentectomy/wedge resection | Not reported | Not available | Not assessable | The review focused on OS, CSS, and RFS, with heterogeneous limited-resection comparisons [22]. |
| Zheng 2020 | Segmentectomy vs lobectomy | Not reported | Not available | Not assessable | The review focused on OS and DFS in clinical T1N0M0 NSCLC [12]. |

**Note:** Pulmonary function outcomes were not uniformly reported across the included systematic reviews and meta-analyses. Most reviews focused primarily on oncologic endpoints such as OS, DFS, RFS, CSS, and recurrence.

Table S10. Surgical-extent classification of included reviews

| Study | Reported comparison | Surgical extent classification | Segmentectomy-only extractable | Wedge-only extractable | Mixed sublobar evidence | Used in segmentectomy-only sensitivity synthesis | Comment |
| --- | --- | --- | --- | --- | --- | --- | --- |
| Bedetti 2017 | Segmentectomy vs lobectomy | Segmentectomy-only | Yes | No | No | Yes | Focused segmentectomy-versus-lobectomy review. |
| Bertolaccini 2022 | Minimally invasive anatomical segmentectomy vs lobectomy | Segmentectomy-only | Yes | No | No | Yes | Minimally invasive anatomical segmentectomy in Stage IA NSCLC. |
| Bertolaccini 2024 | Segmentectomy vs lobectomy | Segmentectomy-only | Yes | No | No | Yes | Segmentectomy-versus-lobectomy review in Stage IA NSCLC; includes RCT and observational evidence. |
| Righi 2023 | Segmentectomy vs lobectomy | Segmentectomy-only | Yes | No | No | Yes | Focused on clinical Stage I NSCLC up to 2 cm; wedge resection not pooled as the main comparison. |
| Winckelmans 2020 | Segmentectomy vs lobectomy | Segmentectomy-only | Yes | No | No | Yes | Segmentectomy-only comparisons; includes Stage I and Stage IA subgroup evidence. |
| Li 2024 | Segmentectomy vs lobectomy | Segmentectomy-only | Yes | No | No | Yes | Segmentectomy-only survival synthesis including OS, DFS, and RFS. |
| Zeng 2020 | VATS segmentectomy vs VATS lobectomy | Segmentectomy-only | Yes | No | No | Yes | Although sometimes described broadly as sublobar resection, the actual comparison was VATS segmentectomy versus VATS lobectomy. |
| Zheng 2020 | Segmentectomy vs lobectomy | Segmentectomy-only | Yes | No | No | Yes | Clinical T1N0M0 NSCLC; segmentectomy-specific evidence extractable. |
| Feng 2021 | Segmentectomy vs lobectomy | Segmentectomy-only | Yes | No | No | No | Survival estimates were reported as ORs rather than HRs; therefore excluded from HR-based sensitivity synthesis. |
| Fatima 2024 | Sublobar resection vs lobectomy | Mixed with extractable subgroups | Yes, subgroup | Yes, subgroup | Yes | Subgroup only | Overall analysis combined sublobar procedures, but segmentectomy and wedge subgroup data were available. |
| Lin 2024 | Segmentectomy/wedge resection vs lobectomy | Mixed with extractable subgroups | Yes, subgroup | Yes, subgroup | Yes | Subgroup only | Directly separated segmentectomy and wedge resection in solid-dominant Stage IA NSCLC. |
| Zhang 2015 JSO | Limited resection including segmentectomy and wedge resection | Mixed with extractable subgroups | Yes, subgroup | Yes | Yes | Descriptive only | Broad historical limited-resection review; substantial overlap with other historical reviews. |
| Liu 2016 | Thoracoscopic sublobectomy vs lobectomy | Mixed with extractable subgroups | Yes, subgroup | Yes, subgroup | Yes | Descriptive only | Overall analysis combined sublobar procedures; subgroup data available but review quality was critically low. |
| Fong 2023 | Sublobar resection vs lobectomy; anatomical segmentectomy sensitivity available | Mixed with extractable subgroups | Yes, sensitivity/subgroup | Partly | Yes | Yes, exploratory | High-quality patient-level meta-analysis, but the overall comparison was not purely segmentectomy-only. |
| Mamede 2024 | Sublobar resection vs lobectomy | Mixed with extractable subgroups | Partly | Partly | Yes | Descriptive only | Segmentectomy and wedge subgroup information was discussed, but exact HR/CI values were not consistently extractable from the main text. |
| Guo 2019 | Sublobar resection vs lobectomy | Mixed with extractable subgroups | Unclear/partly | Unclear | Yes | No | Direction and subgroup extraction were not sufficiently clear for quantitative HR-based sensitivity synthesis. |
| Lv 2021 | Sublobectomy vs lobectomy | Mixed sublobar | No | No | Yes | No | No segmentectomy-specific effect was extractable; overall comparison was mixed sublobectomy. |
| Zhang 2015 ATS | Segmentectomy vs lobectomy | Segmentectomy-only | Yes | No | No | Yes | Anatomical segmentectomy-versus-lobectomy review; historical primary-study overlap likely. |

**Note:** Surgical-extent classes were defined as segmentectomy-only, mixed with extractable subgroups, mixed sublobar, or unclear. Reviews described only as “sublobar resection,” “limited resection,” or “sublobectomy” were not interpreted as segmentectomy-specific unless segmentectomy data were separately extractable.

Table S11. Deduplication and overlap adjudication for updated pooled analyses

| Domain | Adjudication rule | Application in this umbrella review | Decision |
| --- | --- | --- | --- |
| Exact duplicate primary study | Match first author, publication year, cohort/source, recruitment period, sample size, stage criteria, surgical comparison, and outcome. | Duplicate primary-study entries reported across multiple meta-analyses were not treated as independent primary-study evidence. | Flag as duplicate or overlapping evidence; retain only one estimate when primary-study-level duplication was identifiable. |
| Same study with multiple outcomes | Treat OS, DFS, RFS, and CSS as distinct outcome domains, but avoid double-counting within the same outcome. | Outcome-specific estimates were summarized separately. OS and DFS/RFS estimates from the same source were not combined as independent evidence for the same endpoint. | Separate by outcome; avoid cross-outcome double-counting. |
| Same study with multiple stage subgroups | Stage I and Stage IA/≤2 cm estimates from the same study were not treated as independent primary studies within the same synthesis. | Stage-specific estimates were retained only for their corresponding stage analysis. | Retain separately by stage when clearly reported; avoid combining overlapping stage strata. |
| Same study with multiple surgical comparisons | Segmentectomy, wedge resection, and mixed sublobar comparisons were adjudicated by surgical extent before quantitative interpretation. | Segmentectomy-only estimates were separated from wedge-containing or mixed sublobar estimates in the surgical-extent synthesis. | Do not interpret all-sublobar pooled estimates as segmentectomy-only evidence. |
| Mixed sublobar definition | Reviews described as “sublobar resection,” “limited resection,” or “sublobectomy” were classified as mixed or unclear unless segmentectomy-specific data were extractable. | Mixed reviews were retained in the broad all-sublobar interpretation, but not treated as direct evidence for anatomical segmentectomy. | Retain for broad all-sublobar evidence; exclude from segmentectomy-only interpretation unless subgroup data are extractable. |
| SEER/NCDB or registry-based overlap | Flag potential patient-population overlap using database source, overlapping calendar years, eligibility criteria, sample size, and stage/surgery definitions. | Registry-based evidence was treated as potentially overlapping when source databases and calendar windows overlapped across reviews. | Residual overlap explicitly acknowledged; strict registry deduplication would require primary-study-level extraction. |
| Highly overlapping reviews | Older reviews sharing many historical primary studies were not considered independent confirmatory evidence. | Historical reviews such as Zhang 2015 JSO, Zhang 2015 ATS, Bedetti 2017, Zheng 2020, and Zeng 2020 were flagged for likely overlap. | Use for transparency and sensitivity interpretation, not as fully independent proof of effect. |
| Historical mixed limited-resection evidence | Older limited-resection evidence may combine non-anatomical wedge resection, anatomical segmentectomy, and varied staging or surgical-era effects. | Broad historical sublobar evidence was interpreted separately from contemporary anatomical segmentectomy evidence. | Discuss as broad historical aggregate evidence with limited direct applicability to modern segmentectomy. |
| Residual overlap risk | Aggregate review-level data cannot fully resolve patient-level overlap across registries, institutions, or reused subgroups. | Residual overlap was acknowledged in Methods, Discussion, and Limitations. | Interpret updated pooled estimates as exploratory aggregate-data syntheses rather than independent patient-level evidence. |

**Note:** Deduplication was based on first author, publication year, cohort or database source, recruitment period, sample size, stage criteria, surgical comparison, and outcome. For registry-based studies, overlapping calendar years and eligibility criteria were used to flag potential patient-population overlap. Because this umbrella review used aggregate data rather than individual patient data, residual overlap could not be fully excluded.

Table S12. Review-level exploratory sensitivity analyses and publication-bias interpretation

| Analysis | Evidence set | k | Main HR (95% CI) | P value | I² (%) | Egger’s test P | Trim-and-fill imputed studies | Trim-and-fill adjusted HR (95% CI) | Leave-one-out HR range | Sensitivity interpretation | Final interpretation |
| --- | --- | --- | --- | --- | --- | --- | --- | --- | --- | --- | --- |
| All-sublobar OS | HR-only, direction-harmonized, review-level all-sublobar estimates | 7 | 1.20 (1.07–1.35) | 0.001 | 58.8 | 0.262 | 1 | 1.18 (1.06–1.32) | 1.18–1.24 | Substantial heterogeneity; Egger’s test did not show clear funnel-plot asymmetry; trim-and-fill imputed one potentially missing estimate. | Exploratory OS evidence; should not be interpreted as definitive superiority evidence. |
| All-sublobar DFS/RFS | HR-only, direction-harmonized, review-level all-sublobar estimates | 5 | 1.20 (1.07–1.34) | 0.001 | 37.7 | 0.028 | 2 | 1.14 (1.02–1.27) | 1.16–1.25 | Small number of review-level estimates; Egger’s test suggested small-study effects; trim-and-fill attenuated the estimate. | Exploratory recurrence-related signal; does not replace primary-study-level deduplicated analysis. |
| All-sublobar Stage I/early-stage OS | All-sublobar estimates; stage separation based on review-level stage labels | 3 | 1.31 (1.14–1.49) | <0.001 | 18.4 | 0.165 | 2 | 1.23 (1.12–1.35) | 1.26–1.47 | Very small number of estimates; Egger’s test should be interpreted cautiously; trim-and-fill attenuated the estimate. | Review-level exploratory sensitivity synthesis only. |
| All-sublobar Stage IA/≤2 cm OS | All-sublobar estimates; stage separation based on review-level stage labels | 4 | 1.13 (0.97–1.31) | 0.125 | 63.6 | 0.800 | 0 | 1.13 (0.97–1.31) | 1.07–1.20 | Substantial heterogeneity; Egger’s test did not suggest clear asymmetry; trim-and-fill did not impute missing estimates. | No clear OS difference in Stage IA/≤2 cm, but interpretation remains exploratory. |
| All-sublobar Stage IA/≤2 cm DFS/RFS | All-sublobar estimates; stage separation based on review-level stage labels | 4 | 1.18 (1.05–1.34) | 0.007 | 44.7 | 0.025 | 1 | 1.14 (1.01–1.30) | 1.14–1.24 | Egger’s test suggested small-study effects; trim-and-fill slightly attenuated the estimate. | Exploratory recurrence-related signal in Stage IA/≤2 cm evidence. |
| Segmentectomy-only OS | HR-only, direction-harmonized segmentectomy-only estimates | 19 | 1.11 (1.03–1.19) | 0.004 | 59.4 | 0.158 | 1 | 1.12 (1.04–1.20) | 1.09–1.13 | Substantial heterogeneity; Egger’s test did not suggest clear asymmetry; trim-and-fill had minimal effect. | Exploratory OS evidence; not definitive because estimates are review-level aggregates. |
| Segmentectomy-only DFS/RFS | HR-only, direction-harmonized segmentectomy-only estimates | 15 | 1.11 (1.04–1.18) | 0.001 | 25.7 | 0.885 | 5 | 1.16 (1.09–1.24) | 1.09–1.13 | Low heterogeneity; Egger’s test did not suggest funnel-plot asymmetry; trim-and-fill imputed five potentially missing estimates. | More stable recurrence-related signal at review-estimate level, but still exploratory. |
| Segmentectomy-only Stage I/early-stage OS | Segmentectomy-only estimates; stage separation based on review-level stage labels | 7 | 1.17 (1.09–1.25) | <0.001 | 30.2 | 0.392 | 1 | 1.17 (1.10–1.26) | 1.14–1.21 | Low-to-moderate heterogeneity; Egger’s test did not suggest clear asymmetry. | Review-level exploratory evidence suggesting modestly higher hazard after segmentectomy in broader Stage I/early-stage populations. |
| Segmentectomy-only Stage I/early-stage DFS/RFS | Segmentectomy-only estimates; stage separation based on review-level stage labels | 6 | 1.15 (1.02–1.30) | 0.023 | 56.2 | 0.936 | 3 | 1.04 (0.90–1.20) | 1.11–1.22 | Moderate heterogeneity; trim-and-fill attenuated the estimate toward the null. | Exploratory; recurrence-related signal was not robust after trim-and-fill adjustment. |
| Segmentectomy-only Stage IA/≤2 cm OS | Segmentectomy-only estimates; stage separation based on review-level stage labels | 11 | 1.06 (0.94–1.19) | 0.372 | 66.0 | 0.704 | 0 | 1.06 (0.94–1.19) | 1.01–1.09 | Substantial heterogeneity, but no significant overall effect; trim-and-fill did not impute missing estimates. | No significant OS difference in segmentectomy-only Stage IA/≤2 cm evidence; exploratory. |
| Segmentectomy-only Stage IA/≤2 cm DFS/RFS | Segmentectomy-only estimates; stage separation based on review-level stage labels | 9 | 1.07 (0.99–1.15) | 0.070 | 0.0 | 0.835 | 0 | 1.07 (0.99–1.15) | 1.06–1.09 | Low heterogeneity and no Egger’s test signal; estimate was close to the null. | No statistically significant DFS/RFS difference in segmentectomy-only Stage IA/≤2 cm evidence; exploratory. |

**Note:** These analyses were based on review-level aggregate estimates and were performed as exploratory sensitivity syntheses. They should not be interpreted as fully deduplicated primary-study-level meta-analyses. HR >1 indicates a higher hazard after sublobar resection or segmentectomy compared with lobectomy. Egger’s test and trim-and-fill results should be interpreted cautiously when the number of estimates is small or heterogeneity is substantial.
